# Enhancement of Exercise Performance by 48 Hours, and 15-Day Supplementation with Mangiferin and Luteolin in Men

**DOI:** 10.3390/nu11020344

**Published:** 2019-02-06

**Authors:** Miriam Gelabert-Rebato, Julia C. Wiebe, Marcos Martin-Rincon, Victor Galvan-Alvarez, David Curtelin, Mario Perez-Valera, Julian Juan Habib, Alberto Pérez-López, Tanausú Vega, David Morales-Alamo, Jose A. L. Calbet

**Affiliations:** 1 Department of Physical Education and Research Institute of Biomedical and Health Sciences (IUIBS), University of Las Palmas de Gran Canaria, Campus Universitario de Tafira s/n, 35017 Las Palmas de Gran Canaria, Spain; miriamgela@hotmail.com (M.G.-R.); marcos.martinrincon@gmail.com (M.M.-R.); victor_galvan@hotmail.es (V.G.-A.); davidcurtelin@gmail.com (D.C.); marioperezvalera@gmail.com (M.P.-V.); totti10amigo@gmail.com (J.J.H.); alberto_perez-lopez@hotmail.com (A.P.-L.); moralesalamo.d@gmail.com (D.M.-A.); 2 Nektium Pharma, Las Mimosas 8, Agüimes, 35118 Las Palmas de Gran Canaria, Spain; jwiebe@nektium.com (J.C.W.); tvega@nektium.com (T.V.); 3 Department of Biomedical Sciences, Faculty of Medicine and Health Sciences, University of Alcalá, Ctra. Madrid-Barcelona, km 33,600, 28871 Alcalá de Henares Madrid, Spain

**Keywords:** sports nutrition, ergogenic aids, polyphenols, performance, O_2_ extraction, ischemia, reperfusion, metabolism, exercise

## Abstract

The natural polyphenols mangiferin and luteolin have free radical-scavenging properties, induce the antioxidant gene program and down-regulate the expression of superoxide-producing enzymes. However, the effects of these two polyphenols on exercise capacity remains mostly unknown. To determine whether a combination of luteolin (peanut husk extract containing 95% luteolin, PHE) and mangiferin (mango leave extract (MLE), Zynamite^®^) at low (PHE: 50 mg/day; and 140 mg/day of MLE containing 100 mg of mangiferin; L) and high doses (PHE: 100 mg/day; MLE: 420 mg/day; H) may enhance exercise performance, twelve physically active men performed incremental exercise to exhaustion, followed by sprint and endurance exercise after 48 h (acute effects) and 15 days of supplementation (prolonged effects) with polyphenols or placebo, following a double-blind crossover design. During sprint exercise, mangiferin + luteolin supplementation enhanced exercise performance, facilitated muscle oxygen extraction, and improved brain oxygenation, without increasing the VO_2_. Compared to placebo, mangiferin + luteolin increased muscle O_2_ extraction during post-exercise ischemia, and improved sprint performance after ischemia-reperfusion likely by increasing glycolytic energy production, as reflected by higher blood lactate concentrations after the sprints. Similar responses were elicited by the two doses tested. In conclusion, acute and prolonged supplementation with mangiferin combined with luteolin enhances performance, muscle O_2_ extraction, and brain oxygenation during sprint exercise, at high and low doses.

## 1. Introduction

Excessive production of reactive oxygen and nitrogen species (RONS) during exercise may cause damage to the cellular structures resulting in maladaptation to exercise [1,2], inflammation [3], muscle [4,5,6] and cardiac fatigue [7], and impairment of executive and cognitive functions [8]. Although some antioxidants may enhance mechanical efficiency (e.g., acetylcysteine) and improve performance [9], they may also counteract some of the signaling processes necessary for the adaptive responses to exercise [10,11,12]. This has prompted the search for products alternative to classical antioxidants capable of modulating redox responses without blunting some beneficial exercise adaptations [13].

Hundreds of natural polyphenols present in edible plants and plant products contribute to the health effects attributed to the consumption of certain foods [14,15,16,17]. Most polyphenols have free radical-scavenging capacity [18], while others act as signaling molecules, or have interesting properties as anti-ageing [19,20], anti-mutagenic [14,21,22] and anti-obesogenic [15,23,24] compounds. After ingestion, some polyphenols can cross the blood-brain barrier and exert specific effects on the central nervous system acting on brain metabolism, neurotransmission, and oxygenation with positive effects on neurogenesis, neurocognitive functions, and mood state [25,26,27]. Some polyphenols may enhance sports performance [28] and facilitate the adaptation to regular exercise by reducing exercise-induced muscle damage [29]. 

During exercise, reactive oxygen and nitrogen species are continuously produced by mitochondrial respiration, but xanthine oxidase (also called xanthine oxidoreductase; XO) and nicotinamide adenine dinucleotide phosphate-oxidase (NADPH oxidase, also called NOX) are also important sources of RONS during exercise [10,30,31]. The classical approach to counteract RONS during exercise has been the administration of antioxidants, i.e., compounds with free radical-quenching properties. This approach has been criticized due to the potential interference with some critical signaling events that depend exclusively on free radicals [32,33,34]. Nevertheless, it has been reported that supplementation with some polyphenolic compounds could avoid some of the adverse effects on performance observed with the intake of antioxidant vitamins, like vitamin C during training [33,34]. Besides, pharmacological inhibition of XO seems to reduce exercise-induced muscle damage both in animals [35] and athletes [36,37].

Natural polyphenols like mangiferin and luteolin are potent antioxidants and inhibitors of XO [23,38,39] and NOX [40,41]. A previous study has shown ergogenic effects after acute supplementation (48 h) with a mango leave extract (MLE, Zynamite^®^) combined with either quercetin or luteolin [27]. No data is available regarding the effects of prolonged mangiferin or luteolin supplementation on exercise performance. Chronic ingestion of either of these two polyphenols could stimulate the antioxidant gene program through up-regulation of the nuclear factor erythroid 2 (NFE2)-related factor 2 (NRF2) transcription factor [39,42] and could elicit an up-regulation of the natural antioxidant enzymes, increasing the capacity of the cells to face the burden of RONS produced during exercise. Mangiferin may also be an excellent bioactive to prevent mitochondrial lipid peroxidation [43], which may occur during prolonged and unaccustomed exercise [30,44]. Moreover, animal experiments indicate that luteolin may down-regulate the expression of the genes (Cyba, Cybb, Ncf1, Ncf4, and Rac2) encoding the enzymatic subunits of NADPH oxidase [40,41]. The selective action of these two polyphenols on XO and NOX is particularly interesting since other sources of free radicals would not be inhibited, permitting the signaling events necessary for the normal adaptation to exercise [34].

Therefore, we hypothesized that the ingestion of these compounds before exercise might help to enhance exercise performance by facilitating mitochondrial respiration through its antioxidant and XO-inhibitory properties, enhancing muscle O_2_ extraction and improving brain oxygenation as previously reported in young women [27].

Consequently, this investigation aimed at determining the acute and prolonged effects of oral supplementation with mangiferin and luteolin botanical extracts on exercise performance, muscle metabolism, and brain and muscle oxygenation in healthy young men. Given the fact that these two polyphenols may have ergogenic effects through several mechanisms, a specific exercise protocol was designed, including phases of low-intensity, high-intensity, and repeated sprinting exercise combined with ischemia-reperfusion episodes.

## 2. Materials and Methods

### 2.1. Subjects

Twelve healthy male physical education students (age = 21.3 ± 2.1 years, height = 176.6 ± 5.8 cm, body mass = 75.7 ± 9.9 kg, body fat = 20.4 ± 5.3%, VO_2_max: 3.69 ± 0.47 L/min and 49.4 ± 8.2 mL/kg/min) agreed to participate in this investigation. The inclusion criteria for participation in the study were: age from 18 to 35 years old; male without chronic diseases or recent surgery; non-smoker; normal resting electrocardiogram; body mass index below 30 and above 18; no history of disease requiring medical treatments lasting more than 15 days during the preceding six months; no medical contraindications to exercise testing; and lack of allergies to peanuts or mango fruit. All volunteers applying for participation met the inclusion criteria. Subjects were requested to avoid intense physical activity 48 h before laboratory tests and to refrain from carbonated, caffeinated and alcohol-containing beverages during the 24 h period preceding the tests. They were also requested to record the dinner preceding the first experimental day and reproduce the same dinner the night before the subsequent experimental days.

All subjects received written and oral information about the experimental procedures before providing their written consent to participate. The study was performed by the Helsinki Declaration and approved by the Ethical Committee of the University of Las Palmas de Gran Canaria (CEIH-2016-02). The sample size required to allow detecting a 5% improvement of performance with a statistical power of 0.8 (α = 0.05), assuming a coefficient of variation for the ergometric test below 5%, was eight subjects. To account for potential dropouts and technical difficulties twelve subjects were finally recruited.

### 2.2. General Procedures

After inclusion, a medical history, resting electrocardiogram, a blood analysis including the assessment of a basic hemogram and general clinical biochemistry tests were carried out to verify the health status of participants. The clinical tests were repeated 24 h and 14 days after the start of supplementation. Subjects were randomly assigned to a placebo (P) or treatment group (T) in a double-blind, counterbalanced crossover design. The placebo group received microcrystalline cellulose capsules of identical aspect containing 500 mg of maltodextrin, while the treatment group received similar capsules containing luteolin and mangiferin. Three subjects were provided with 50 mg/day of peanut husk extract containing 95% luteolin and 140 mg/d of MLE (Zynamite^®^) containing 100 mg/day of mangiferin (low-dose treatment group; L), while the remaining three subjects of the treatment group received 100 mg/day of peanut husk extract containing 95% luteolin and 420 mg/day MLE containing 300 mg/day of mangiferin (high-dose treatment group; H). A detailed description of the composition of the two supplements can be found elsewhere [27]. Subjects ingested the supplements every eight hours during 15 days, then after 3–4 weeks of washout, treatment groups received placebo, and the placebo group was again split into low and high-dose treatment subgroups, also for 15 days. The low dose of mangiferin was based on a pharmacokinetic study by Hou et al. [45] showing oral absorption and mean residence time close to 7 h, after the ingestion of 0.1 g of pure mangiferin in humans. The high dose of luteolin was based on human pharmacokinetic data obtained following the ingestion of an artichoke leaf extract rich in luteolin [46], and 100 mg of encapsulated luteolin [47], as previously reported [27].

Subjects reported to the laboratory early in the morning after a 12 h fast, 48 h after the start of the supplementation, and received an extra dose of the assigned supplements. After that, their body composition was determined using dual-energy X-ray absorptiometry (Lunar iDXA, General Electric, WI, USA), followed by the assessment of their resting metabolic rate (RMR) by indirect calorimetry (Vyntus CPX; Jaeger-CareFusion, Hoechberg, Germany) during 20 min lying supine and motionless on a comfortable stretcher while a quiet environment was maintained. Then near-infrared spectroscopy (NIRS) optodes were placed on the frontal lobe and the musculus vastus lateralis and medialis as previously reported [48,49]. With the subjects resting supine a 10 cm wide cuff connected to a rapid cuff inflator (SCD10, Hokanson, Bellevue, DC, USA) was placed around the right thigh, as proximal as possible, as previously reported [49]. After an initial 3 min period with legs elevated on a cushion, the cuff was inflated at 300 mmHg at maximal speed, resulting in full occlusion of the legs’ circulation within less than 2 s, which was maintained for 8 min. At the end of the occlusion period, the cuff was released and the hyperemic response measured during the next 2 min.

### 2.3. Exercise Protocol

**Figure 1:**
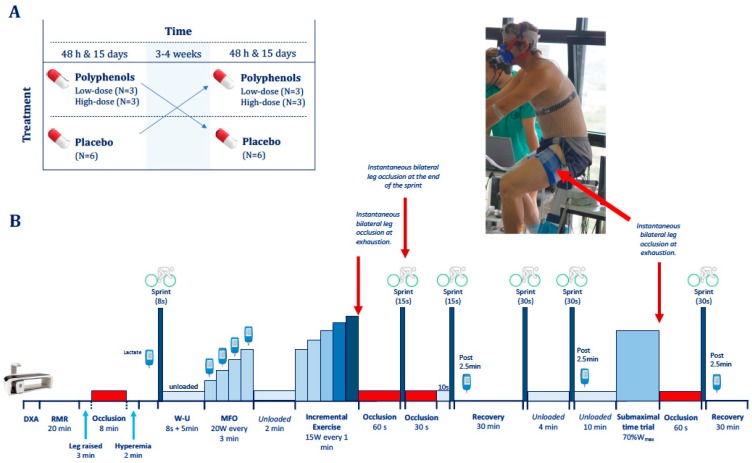
Experimental protocol. (**A**) Botanical extracts of mangiferin and luteolin were administered during following a double-blind, counterbalanced crossover design. (**B**) Exercise protocol. Forty-eight hours after the start of the supplementation subjects reported to the laboratory and their body composition and resting metabolic rate (RMR) were determined. The exercise protocol started with a warming-up 8 s isokinetic sprint on a cycle ergometer, followed by 5 min of unloaded pedaling (∼40 rpm) and an incremental exercise test (20 W/3 min) to determine their maximal fat oxidation (MFO). This was followed by 2 min of unloaded pedaling and an incremental exercise test (15 W/min) until exhaustion to determine the VO_2_max. At exhaustion, ischemia was applied to both legs for 60 s. Then the cuff was released, and the subjects sprinted maximally for 15 s. At the end of the 15 s sprint, a second occlusion was started for 30 s, which was followed by 10 s of free circulation with unloaded pedaling and a second 15 s sprint. Then the subjects rested for 30 min and after that performed two 30 s Wingate tests interspaced by 4 min of unloaded pedaling. After 10 min of unloaded pedaling a submaximal constant-intensity time trial to exhaustion was started at 70% of the intensity reached at exhaustion in the incremental exercise test (Wmax). At the end of the endurance test, ischemia was instantaneously applied for 60 s, followed by the last Wingate test with open circulation and 30 min of recovery on a stretcher. Blood samples for blood lactate assessment were obtained as indicated in the figure. This protocol was repeated after 15 days of supplementation.

The exercise protocol (Figure 1) started with a warm-up consisting of 8 s of isokinetic sprint on a cycle ergometer (Excalibur Sport 925900, Lode, Groningen, The Netherlands) (Figure 1). This was followed by a 5 min recovery period during which the subjects pedaled at low speed (~40 rpm) with no load. Next, an incremental exercise test was performed to determine the maximal fat oxidation capacity (MFO) (see below). The MFO test was followed by 2 min of unloaded pedaling, and then the load was increased to the same intensity reached at the end of the MFO test and increased 15 W every min until exhaustion to determine the VO_2_max. Immediately upon exhaustion, the cuffs were instantaneously inflated at maximal speed and pressure (i.e., 300 mmHg) to completely occlude the circulation (ischemia) for 60 s, as previously reported [49]. The subjects remained seated and quiet on the cycle ergometer without pedaling during the periods of ischemia. At the 50th second of ischemia, a 10 s countdown was started while the subjects got ready to sprint as fast and hard as possible for 15 s. At the start of the sprint, following the 60 s of ischemia, the cuff was instantaneously deflated such that the sprint was carried out with the circulation opened. At the end of the 15 s sprint, a second occlusion was started for 30 s, which was followed by 10 s of free circulation. At the end of the 15 s sprint, a second occlusion was started for 30 s, then the cuff was released and the subjects pedaled slowly at 20 W while a 10 s countdown towards a second 15 s sprint was started. Thus, the second 15 s sprint was carried out after a cycle of ischemia (30 s) followed by 10 s reperfusion. Then, after 2.5 min of passive recovery on the bike, a blood sample was obtained from the earlobe to measure blood lactate concentration (Lactate Pro 2, Arkray, Kyoto, Japan). After the second 15 s sprint, the volunteers rested for 30 min. During the first 20 min they rested lying on a stretcher; then, they moved back to the ergometer for unloaded pedaling at low speed while the instruments were reconnected. At the completion of the 30 min recovery, a Wingate test (sprint lasting 30 s) was performed followed by a 4 min recovery period during which the subjects pedaled at low speed with the cycle ergometer unloaded. At the end of this short recovery, a second Wingate test was performed. The second Wingate was followed by a 10 min recovery with slow pedaling at 20 W. After 2.5 min of slow unloaded pedaling on the cycle ergometer, a blood sample was obtained from the earlobe to measure blood lactate concentration. At the completion of the 10 min recovery period, a submaximal constant-intensity time trial to exhaustion was started at 70% of the intensity reached in the incremental exercise test (Wmax). In control experiments, with the subjects rested before the time trial, our volunteers were able to sustain this intensity for 20–60 min, depending on their fitness status. This test was used to assess the effects of the supplements on endurance capacity, since the test likely started with very low glycogen levels, replicating the conditions of the final stages of most endurance competitions. At the end of the endurance test (exhaustion), the circulation of both legs was occluded again for 60 s. At the 50th second of ischemia, a 10 s countdown was started while the subjects prompted to perform a final Wingate (30 s) sprint. At the end of this sprint, the subjects remained seated on the bike while pedaling at low speed with the cycle ergometer unloaded. After 2.5 min of recovery, another blood sample was obtained from the earlobe to measure blood lactate. Then the subjects moved to the stretcher and rested until reaching 30 min of recovery. Strong verbal encouragement was provided throughout the entire exercise protocol and particularly approaching task failure and during the sprints.

This exercise protocol was repeated after 15 days of supplementation, to determine potential effects due to prolonged supplementation. After 3–4 weeks of washout, the acute and chronic phases were repeated following the crossover counterbalanced design described above.

### 2.4. Power Output and VO_2_max

All sprints were performed with the cycle ergometer set in isokinetic mode and results reported as instantaneous peak power (PPO) and mean power output (MPO) [49]. Oxygen uptake was measured with a calibrated metabolic cart (Vyntus CPX; Jaeger-CareFusion, Hoechberg, Germany). Respiratory variables were analyzed breath-by-breath and averaged every 5 s during the sprints. During maximal exercise 15-breath, rolling averages were generated starting from 120 s before the end of the exercise, and the highest 15-breath averaged value was taken as the VO_2_max.

### 2.5. Maximal Fat Oxidation

This test started at 20 W for 3 min, followed by 20 W increases every 3 min until the respiratory exchange ratio (RER) was ≥1.0 [50,51]. The VO_2_ and VCO_2_ data averaged during the last min of each load, and was used to determine the maximum rate of fat oxidation as previously reported [50,51]. Blood lactate concentrations were determined from earlobe samples obtained after 90 s after each increase in intensity.

### 2.6. Exercise Efficiency, Supramaximal Exercise O_2_ Demand, and Oxygen Deficit

The O_2_ demand during the sprints was calculated from the linear relationship between the last 60 s averaged VO_2_ of each load, measured during the MFO and the exercise intensity. The accumulated oxygen deficit (AOD), representing the difference between O_2_ demand and the actual VO_2_, was determined as previously reported [52,53]. The delta energy efficiency of exercise was determined as the slope of the linear relationship between work and energy expenditure [54], using the data collected during the MFO tests.

### 2.7. Cerebral and Musculus Vastus Lateralis Oxygenation

Cerebral oxygenation was assessed using near-infrared spectroscopy (NIRS, NIRO-200 NX, Hamamatsu, Hamamatsu City, Japan) employing spatially resolved spectroscopy to obtain the tissue oxygenation index (TOI) using a pathlength factor of 5.92 [55]. The first NIRS optode was placed on the right frontoparietal region at 3 cm from the midline and 2–3 cm above the supraorbital crest, to avoid the sagittal and frontal sinus areas [56]. This optode placement allows recording the tissue oxygenation of the superficial frontal cerebral cortex, which may influence exercise performance [57,58]. A second optode was placed in the lateral aspect of the thigh at middle length between the patella and the anterosuperior iliac crest, over the middle portion of the musculus vastus lateralis and an additional optode was placed on the vastus medialis at 1/8 distance between the iliac spine and the joint space in front of the medial ligament. The quadriceps muscle oxygenation index (TOI) was obtained from the average of the mean TOI of the two vastus.

### 2.8. Diet Analysis

Subjects’ dietary information was collected using dietary logs during four days, including one weekend day, on two occasions: before the start of the supplementation, and after one week into each supplementation period, using dietary logs. For this purpose, subjects were provided with a dietary diary and a kitchen scale (1 g precision from 0 to 5000 g, calibrated in our laboratory with Class M1 calibration weights, Schenk) and instructions to report in grams all food and drinks ingested. The information recorded was later analyzed with specific software for the Spanish diet (Dial, Alce Ingeniería, Madrid, Spain [59]).

### 2.9. Statistics

Variables were checked for normal distribution by using the Shapiro-Wilks test. When necessary, the analysis was carried out on logarithmically transformed data. A three-way repeated-measures ANOVA test with time (two levels: 48 h and 15 days), treatment (two levels: placebo and polyphenol treatment) and polyphenols dose (two levels: low and high) as between-subjects factors was first applied. Pairwise comparisons were carried using the least significant post hoc test (LSD). The relationship between variables was determined using linear regression analysis. Values are reported as the mean ± standard error of the mean (unless otherwise stated). *p* ≤ 0.05 was considered significant. Statistical analysis was performed using SPSS v.15.0 for Windows (SPSS Inc., Chicago, IL, USA).

## 3. Results

Polyphenols had no significant effects on the clinical blood biochemistry and hemogram tests (Tables S1 and S2). The diet was not significantly altered by the treatment regarding total energy, macronutrients, vitamins, dietary fiber, and plant sterols intakes. Likewise, no significant alterations were observed in body weight or resting metabolic rate, resting blood pressure, blood lactate concentration or heart rate after polyphenols administration (Table S3). The level of deoxygenation reached during the occlusion performed at rest was similar in all conditions, as well as the increase in tissue oxygenation index elicited by the post-ischemic hyperemia.

### 3.1. Incremental Exercise Test

All respiratory variables responded similarly to the placebo and the polyphenol treatments. As reflected in Table 1, the subjects exercised to a similar extent in all tests. Neither the VO_2_max nor the load reached at exhaustion (Wmax) were affected by the treatment. There was a slight 2 mmHg improvement in P_ET_O_2_ in the second test which was also accompanied by a small reduction in P_ET_CO_2_ (~2 mmHg), without differences due to the supplementation administered.

Lactate responses to submaximal exercise were almost identical. Although blood lactate concentration at 200 W was 11% lower after the polyphenol treatment, this effect did not reach statistical significance (*p* = 0.11) (Table 1). Delta efficiency was transiently improved 48 h after the start of polyphenols in the group receiving the lower dose (compared to placebo, *p* = 0.002, ANOVA treatment × time × dose interaction *p* = 0.001). Polyphenols supplementation did not alter the MFO nor peak HR (Table 1).

### 3.2. Sprint Exercise after Ischemia-Reperfusion

**Figure 2:**
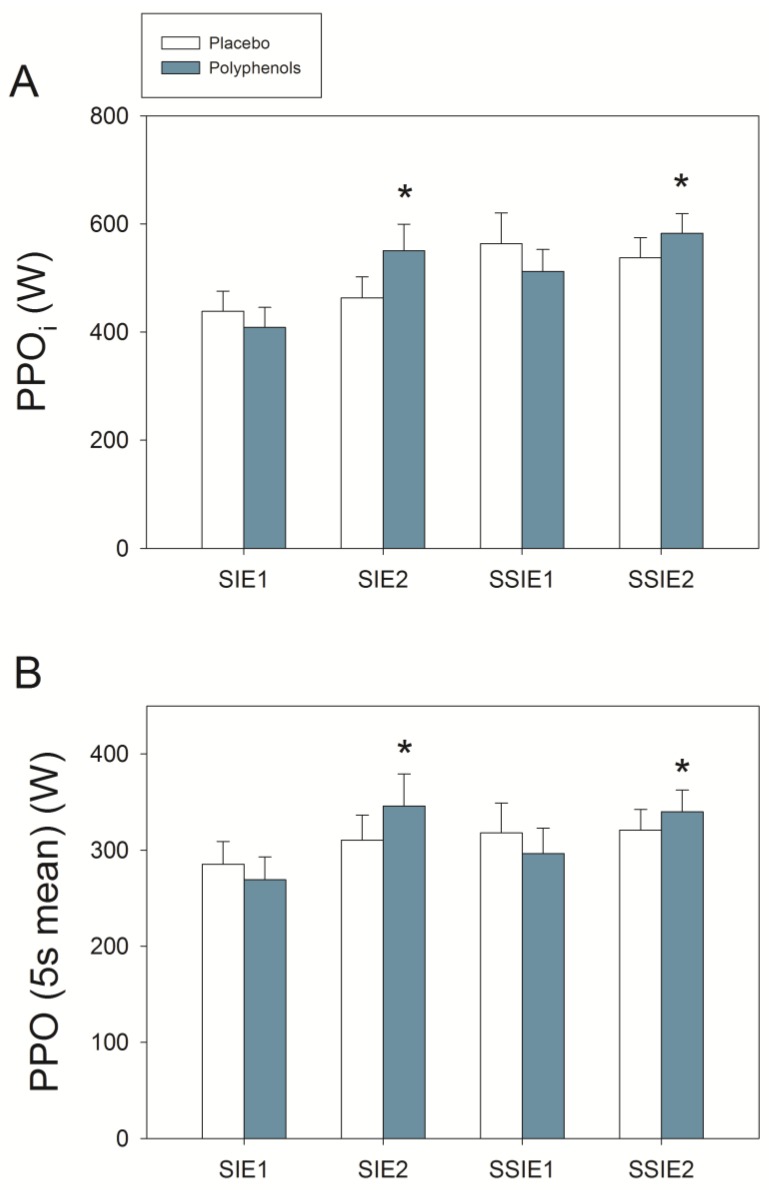

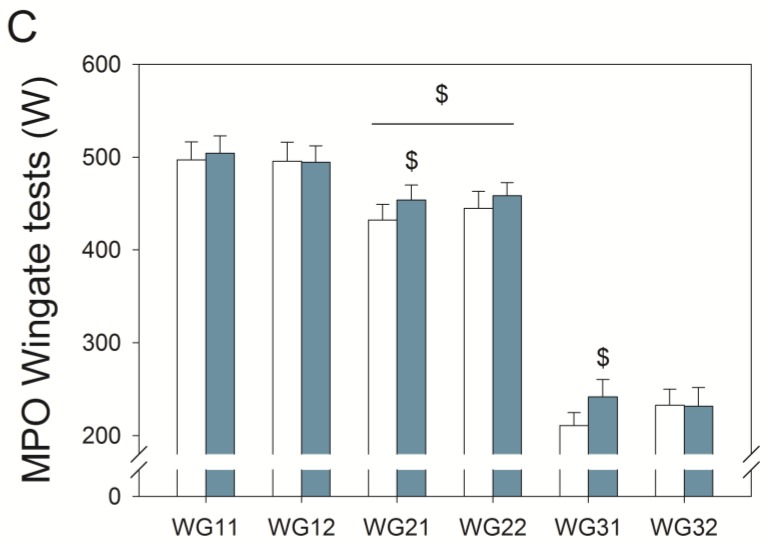
Performance during the sprint exercise after the ingestion of polyphenols (mangiferin + luteolin) or placebo. (**A**) Peak power output in 15 s sprints performed after ischemia. (**B**) Mean power output during the first 5 s during the sprints performed after ischemia. SIE: first sprint after incremental exercise, SSIE: second sprint after incremental exercise. Number 1 indicates after 48 h and 2 after 15 days of supplementation. (**C**) Mean power output during the 30 s Wingate test. WG: Wingate test, the first number represents the Wingate order number (1, 2, or 3), the second number (1 or 2) indicates after 48 h and 2 after 15 days of supplementation, respectively. * *p* < 0.05 compared with 48 h test in the same condition. $ *p*< 0.05 for treatment effect. ANOVA Wingate × time × treatment interaction, *p* = 0.027. N = 12.

The PPO was not altered by the acute administration of polyphenols (Figure 2A). Following fifteen days of supplementation, PPO in the sprints preceded by ischemia was 500.0 ± 120.1 and 566.4 ± 141.9 W, in the placebo and polyphenol trials, respectively (*p* = 0.11). Nevertheless, from the first (48 h) to second trial (15 days), PPO was enhanced by 22% when the subjects were taken polyphenols (*p* < 0.05), being this effect more marked in the first (+31%) than the second sprint (+14%) (first sprint compared with the second sprint, *p* < 0.05; ANOVA sprint × trial × treatment × dose interaction *p* = 0.026). There were no significant differences between the higher and lower doses of polyphenols on PPO.

In the sprints post-ischemia performed with polyphenols, the MPO developed during the first 5 s was increased by 23% from 48 h to 15 days (272.5 ± 63.8 and 333.8 ± 93.2 W, respectively, *p* = 0.01). In contrast, no significant changes were observed from 48 h to 15 days in the placebo conditions (Figure 2B).

Despite the fact that the mean power output remained at the same level (256 ± 56 and 268 ± 75 W, in the placebo and mangiferin + luteolin condition, respectively, *p* = 0.45), the mean VO_2_ during the sprints post-ischemia was reduced by 5.7% after the administration of mangiferin + luteolin (from 666 ± 98 to 628 ± 77 mL, in the placebo and mangiferin + luteolin conditions, respectively, *p* = 0.010) (Table 2). Although the O_2_ deficit was 23% larger after the ingestion of mangiferin + luteolin, this difference was not statistically different (*p* = 0.245). The peak blood lactate measured 2.5 min after the last sprint postischemia was unchanged in the placebo experiments (9.8 ± 2.7 and 10.4 ± 2.1 mM, *p* = 0.35), but increased from 9.5 ± 2.5 to 11.4 ± 1.8 mM (48 h and 15 days, respectively) after the ingestion of polyphenols (*p* = 0.04) (Table 1).

### 3.3. Wingate Tests

**Figure 3:**
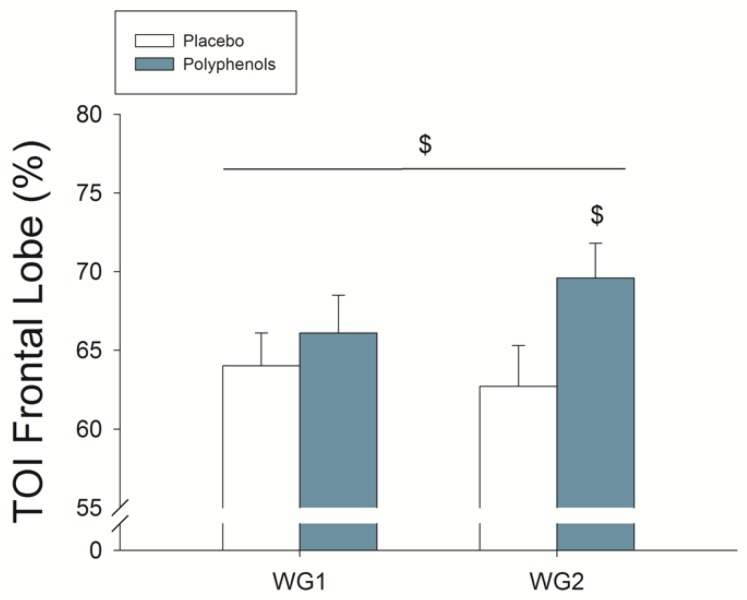
Frontal lobe oxygenation index (TOI) during the first two 30 s Wingate tests after the ingestion of polyphenols (luteolin + mangiferin) or placebo. Number 1 indicates after 48 h and 2 after 15 days of supplementation. $ *p* < 0.05 for treatment effect. N = 12.

**Figure 4:**
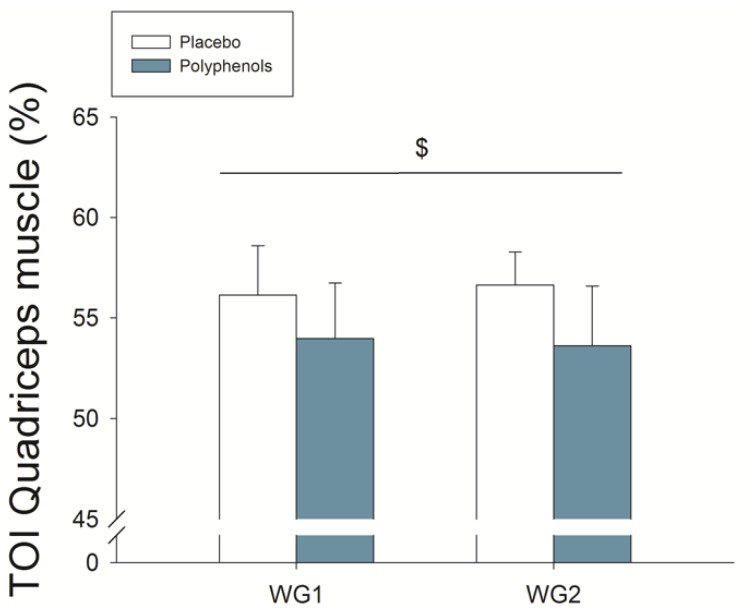
Quadriceps muscle oxygenation index (TOI, mean of the *musculus vastus lateralis* and *vastus medialis*) during the first two 30 s Wingate tests after the ingestion of polyphenols (mangiferin + luteolin) or placebo. Number 1 indicates after 48 h and 2 after 15 days of supplementation. $ *p*< 0.05 for treatment effect. N = 12.

Compared to placebo, polyphenol intake resulted in 4.0% greater MPO (48 h and 15 days assessments combined, *p* = 0.017; ANOVA Wingate × time × treatment *p* = 0.027). Acutely, compared to placebo, polyphenol administration enhanced MPO by 5% in the second Wingate test (*p* = 0.009) (Figure 2C). This was accompanied by enhanced brain oxygenation (Figure 3) (ANOVA treatment effect *p* = 0.02), being this response greater for the higher dose (ANOVA, treatment × dose interaction *p* = 0.047). Quadriceps muscle oxygenation index during sprint exercise was significantly lower, reflecting enhanced O_2_ extraction, after the ingestion of polyphenols both after 48 h (59.7 ± 6.0 and 57.9 ± 6.4%, *p* = 0.007) and 15 days (60.1 ± 3.9 and 57.0 ± 6.1%, *p* = 0.007) supplementation (ANOVA, treatment × dose interaction *p* = 0.01) (Figure 4). Oxygen uptake during the sprints was 6.0% lower after the ingestion of mangiferin + luteolin (*p* = 0.010) (Table 3). Neither the heart rate nor respiratory variables were significantly altered by the ingestion of polyphenols during the two Wingate tests (Table 3).

The last sprint was performed after a time trial to exhaustion followed by a 60 s of ischemia, in a situation of extreme fatigue and low-availability of energy resources. After 48 h of supplementation, MPO was 15% higher in the group receiving polyphenols than in the placebo group (*p* = 0.04). No significant differences were observed neither in brain oxygenation index during the last Wingate test (65.8 ± 8.6 and 68.5 ± 7.2%, for the placebo and polyphenols trials, respectively, *p* = 0.38) nor in quadriceps muscle oxygenation index (57.1 ± 6.7 and 55.8 ± 9.0%, for the placebo and polyphenols trials, respectively, *p* = 0.22). Neither there was a significant difference in the mean lactate responses after incremental exercise nor after the three Wingate tests (10.3 ± 2.4 and 11.1 ± 2.3 mM, for the placebo and polyphenols trials, respectively, *p* = 0.15).

### 3.4. Final Time Trial

No significant effects were observed in the total work performed during the final time trial (101.3 ± 56.6 and 103.5 ± 61.6 kJ, for the placebo and polyphenol trials, respectively, *p* = 0.85). Although the brain oxygenation index was higher after the ingestion of polyphenols, this difference did not reach statistical significance (64.6 ± 6.5 and 68.0 ± 6.0%, for the placebo and polyphenol trials, respectively, *p* = 0.18). The quadriceps muscle oxygenation index was not significantly altered during the final time trials (61.3 ± 6.3 and 60.6 ± 8.5%, for the placebo and polyphenol trial, respectively *p* = 0.34).

### 3.5. Quadriceps Muscle O_2_ Extraction during Ischemia

During the first five seconds of the occlusion, the quadriceps muscle oxygenation index was reduced to lower levels after the ingestion of polyphenols (*p* = 0.04) (Figure 4).

## 4. Discussion

This study shows that a mango leaf extract rich in mangiferin in combination with luteolin enhances exercise performance during sprint exercise and facilitates muscle oxygen extraction. In addition, this polyphenolic combination improves muscle performance after ischemia-reperfusion by three main mechanisms. Firstly, it facilitates muscle oxygen extraction as demonstrated by the greater reduction of the muscle oxygenation index during the first five seconds of total occlusion of the circulation at exhaustion. Secondly, it reduces oxygen consumption during the sprints preceded by ischemia. Thirdly, it may have facilitated ATP production through additional recruitment of the glycolysis, as indicated by the higher levels of blood lactate concentration observed in the sprints performed after ischemia-reperfusion. Importantly, mangiferin + luteolin enhanced mean power output during prolonged sprints (30 s Wingate test) carried out after 30 min of recovery following an incremental exercise test. This improvement in prolonged sprint performance was accompanied by enhanced brain oxygenation and larger muscle oxygen extraction during the sprints.

### 4.1. A Combination of Mangiferin and Luteolin Botanical Extracts Improves Muscle O_2_ Extraction

Although it is well established that increasing O_2_ delivery enhances performance during whole body incremental exercise to exhaustion as well as during submaximal aerobic exercise [60,61,62], performance is not limited by muscle oxygen delivery during a single sprint exercise, at least in healthy humans exercising at sea level [63]. Although O_2_ delivery has not been measured during repeated sprint exercise in humans, muscle biopsy metabolite data [64,65,66] and whole body VO_2_ assessments [52,67] indicate a greater dependency on aerobic metabolism during high-intensity intermittent exercise to exhaustion. Therefore, reducing the need for O_2_ may be advantageous for performance during repeated sprint exercise. 

In the present investigation, we have shown that mangiferin + luteolin supplementation allows the skeletal muscle to reach lower levels of tissue oxygenation during sprint exercise and post-exercise ischemia. This effect could be explained by a better microvascular distribution of perfusion (prioritizing the active skeletal muscle fibers) [68,69] and enhanced mitochondrial O_2_ extraction. The most plausible mechanism by which mangiferin + luteolin supplementation could have enhanced O_2_ extraction is by improving mitochondrial bioenergetics [70], which could be impaired by the high levels of reactive oxygen and nitrogen species (RONS) produced during repeated sprint exercise [2,10,71].

Lower muscle perfusion after the administration of mangiferin + luteolin is unlikely because the polyphenols effects on muscle extraction were greater during the second Wingate test, i.e., when skeletal muscle blood flow is expected to increase faster and to a higher level [52,72]. Moreover, the fact that the heart rate response was not different with supplementation also argues against a different cardiovascular regulation between conditions. The matching between tissue perfusion and VO_2_ at the microvascular level cannot be assessed with current technology during whole body exercise in humans [73] and will not be further discussed here.

### 4.2. A Combination of Mangiferin and Luteolin Botanical Extracts Enhances Sprint Performance after Ischemia-Reperfusion

In agreement with our previous study, performance was improved in the sprints carried out immediately after ischemia (first 15 s sprint). The effect was less marked during the second 15 s sprint, which was preceded by 30 s of ischemia and 10 s of active recovery with reoxygenation [27]. The latter, combined with the greater level of muscle deoxygenation during the first 5 s of ischemia in the experiments performed with polyphenols (Figure 4), suggests that when the PO_2_ is very low, as expected when ischemia is applied after maximal exercise [49], mitochondrial bioenergetics is likely enhanced by the administration of mangiferin + luteolin. This observation concurs with animal studies showing that luteolin [74,75,76,77,78] and mangiferin [79] attenuate the ischemia-reperfusion injury in different tissues. This protective effect of both polyphenols has been attributed to their potent direct free-radical scavenging properties and their inhibitory action on the superoxide-generating enzymes XO and NOX, which are activated during sprint exercise [10] and ischemia-reperfusion [76,79,80,81].

During high-intensity exercise as well as during ischemia, nitric oxide (NO) is produced in skeletal muscle from nitrite by the action of nitrite reductases such as myoglobin [82,83], deoxyhemoglobin [84] and XO [80,85]. Xanthine oxidoreductase usually reduces molecular oxygen to superoxide, but at low oxygen tensions and pH, as observed during prolonged sprints [48,86], repeated sprints [66] and post-exercise ischemia [49], this enzyme can also reduce nitrite to NO [80]. The NO formed can bind to cytochrome c oxidase of the mitochondrial electron transport chain, reducing electron flow and oxygen utilization [87]. Thus, in this investigation, the potential inhibitory action of mangiferin + luteolin on XO might have been beneficial during high-intensity exercise, ischemia and ischemia-reperfusion by reducing superoxide and secondary RONS generation, and attenuating NO production from nitrite in skeletal muscle. Consequently, mangiferin + luteolin could have facilitated mitochondrial respiration and aerobic energy production during the sprints and ischemia periods, as indicated by the lower levels of muscle oxygenation observed here when the ingestion of polyphenols preceded the sprints. At the same time, mangiferin + luteolin could have facilitated mitochondrial bioenergetics, improving muscle efficiency during high-intensity exercise [88].

### 4.3. A Combination of Mangiferin and Luteolin Botanical Extracts Increases Frontal Lobe Oxygenation during Repeated Sprint Exercise

Given the high sensitivity of the brain to hypoxia [89], any small reduction of brain oxygen delivery could potentially alter brain functioning and contribute to fatigue. Moreover, reduced brain oxygenation may facilitate local production of RONS, which may combine with circulating RONS released by contracting muscles, particularly during high-intensity exercise [90]. This could also deteriorate cognitive and executive function during exercise, reducing performance in complex tasks [91,92]. Thus, it is not surprising that the reduction in brain oxygenation has often been argued as a mechanism lowering exercise performance [48,58,93,94,95]. Moreover, fatigue can be swiftly relieved by raising the FiO_2_, during exercise in severe acute hypoxia [94].

In agreement with our previous study [27], the ingestion of mangiferin + luteolin improved frontal lobe oxygenation during the prolonged sprints. This effect may be related to a better distribution of blood flow between tissues or enhanced cerebral vasodilation facilitated by the polyphenols [96]. During sprint exercise, the PaCO_2_ is markedly reduced what may cause vasoconstriction in the brain circulation [97]. The latter combined with the increased production of RONS during sprint exercise, which may hamper endothelial NO production and NO bioavailability, could contribute to reducing brain perfusion and oxygenation. Mangiferin + luteolin supplementation could have improved brain oxygenation during sprint exercise likely through its antioxidant properties, inhibitory action on endothelial NOX [81], suppressive effects on the endoplasmic reticulum-induced stress [96], and increasing the bioavailability of vascular NO [98].

Although the improvement in performance reported here may seem small it is superior to that reported for caffeine during repeated Wingate tests [99]. Moreover, the smallest yet meaningful change in performance for elite male cyclists is as little as 1%, which is difficult to detect in single studies because of the typical measurement error (i.e., 0.7–4.7% [100]. Thus, the improvements elicited by mangiferin + luteolin in peak and mean power output may be critical in sports disciplines where sprint performance in state of fatigue may decide the winner [101].

### 4.4. Limitations

Although the effects on performance, O_2_ extraction, and cerebral oxygenation were robust, this study is limited by the relatively small sample and lack of oxidative stress biomarkers assessment. Although women were not recruited in this investigation, we have previously shown improvement of sprint performance and brain oxygenation in men and women after 48 h supplementation with mangiferin combined with either luteolin or quercetin [27].

Excessive RONS production may cause muscle damage [1,2], fatigue [30] and maladaptation. However, it is thought that exercise-induced RONS act like a hormetic signal necessary for an optimal adaptation to exercise training [102]. According to the hormesis theory, ingestion of antioxidants before exercise may blunt RONS-mediated signaling needed for adaptation [32,102]. However, the use of antioxidants during high-intensity training sessions could allow withstanding high-stress training sessions [1,67], displacing the bell-shaped hormesis curve to higher intensities [102]. Although we have identified some physiological mechanisms, whether the ingestion of mangiferin combined with luteolin could facilitate the adaptive response to high-intensity training remains unknown. Future studies using muscle biopsies are needed to examine whether mangiferin and luteolin modulate RONS induced signaling or prevent oxidative stress.

## 5. Conclusions

Supplementation with the combination of two botanical extracts of mangiferin and luteolin enhances exercise sprint performance, likely by improving brain oxygenation and allowing a higher muscle extraction of oxygen. These effects were observed following 48 h and 15 days of supplementation without significant differences between the two doses tested.

## Figures and Tables

**Table 1:** (a) Effects of mangiferin and luteolin botanical extracts on muscle energy efficiency, heart rate, performance, and pulmonary gas exchange during incremental exercise to exhaustion. (b) Effects of mangiferin and luteolin botanical extracts on muscle energy efficiency, heart rate, performance, and pulmonary gas exchange during incremental exercise to exhaustion and the final time trial.

**(a)**																	
		**Placebo (48 h)**			**Placebo (15 Days)**			**MA + Luteolin (48 h)**			**MA + Luteolin (15 Days)**			**Treatment**	**Pre-Post**	**T × t**	**T × t × d**
Delta Efficiency (%)	L	27.0	±	2.5	29.2	±	4.6	30.0	±	2.1	27.2	±	1.9	0.74	0.73	0.46	<0.001
	H	28.9	±	2.1	28.0	±	1.9	27.1	±	2.1	29.7	±	3.4				
MFO (mg/min)	L	392.2	±	40.0	347.3	±	53.6	393.7	±	100.9	370.9	±	52.9	0.81	0.35	0.50	0.84
	H	399.8	±	129.0	367.3	±	107.4	377.7	±	143.6	385.8	±	178.4				
MFO VO_2_ (mL/min)	L	1377	±	282	1260	±	136	1260	±	173	1313	±	205	0.58	0.17	0.75	0.33
	H	1455	±	406	1389	±	301	1478	±	385	1387	±	455				
Wmax (W)	L	277	±	30	282	±	25	288	±	25	271	±	24	0.87	0.11	0.16	0.03
	H	291	±	48	286	±	42	291	±	48	291	±	47				
HRmax (beats/min)	L	192	±	8	187	±	14	187	±	12	192	±	8	0.20	0.33	0.13	0.08
	H	193	±	8	189	±	10	198	±	10	194	±	12				
VO_2_max (mL/min)	L	3568	±	513	3660	±	318	3649	±	387	3623	±	240	0.87	0.11	0.16	0.026
	H	3821	±	456	3742	±	566	3770	±	590	3681	±	567				
RERmax	L	1.17	±	0.09	1.16	±	0.05	1.18	±	0.06	1.14	±	0.03	0.2	0.33	0.13	0.08
	H	1.11	±	0.03	1.14	±	0.04	1.13	±	0.07	1.12	±	0.05				
V_E_max (L/min)	L	148	±	35	161	±	24	153	±	27	167	±	38	0.78	0.61	0.47	0.54
	H	161	±	21	167	±	25	164	±	20	160	±	17				
BFmax (breaths/min)	L	56	±	13	63	±	11	60	±	10	64	±	15	0.90	0.67	0.026	0.86
	H	62	±	9	64	±	11	63	±	8	64	±	8				
P_ET_CO_2_ (mmHg)	L	37.1	±	2.9	33.3	±	4.8	37.3	±	3.3	34.2	±	3.5	0.69	0.07	0.63	0.57
	H	33.5	±	2.6	31.8	±	4.6	32.8	±	2.7	33.1	±	2.2				
P_ET_O_2_ (mmHg)	L	117	±	5	119	±	4	117	±	4	120	±	6	0.47	0.08	0.61	0.91
	H	119	±	3	119	±	3	119	±	3	118	±	2				
**(b)**																	
		**Placebo (48 h)**			**Placebo (15 days)**			**MA + Luteolin (48 h)**			**MA + Luteolin (15 days)**			**Treatment**	**Pre-Post**	**T × t**	**T × t × d**
Lact at 100 W (mM)	L	1.9	±	0.5	1.8	±	0.5	1.7	±	0.3	1.9	±	0.4	0.55	0.94	0.41	0.92
	H	2.1	±	1.1	2.1	±	1.3	2.1	±	1.0	2.1	±	1.6				
Lact at 200 W (mM)	L	5.8	±	2.6	6.0	±	1.6	5.2	±	1.2	5.7	±	0.5	0.11	0.59	0.44	0.69
	H	6.4	±	3.9	5.9	±	3.2	5.0	±	1.9	5.4	±	3.7				
LT 4 mM (W)	L	177	±	29	173	±	26	181	±	19	170	±	8	0.78	0.40	0.84	0.39
	H	180	±	58	177	±	63	181	±	48	182	±	68				
Lact Peak Post-Ischemia (mM)	L	9.1	±	2.2	10.2	±	1.5	8.6	±	2.4	11.2	±	1.2	0.53	0.02	0.29	0.88
	H	10.5	±	3.2	10.6	±	2.7	10.4	±	2.4	11.7	±	2.3				
RPE (post Incremental exercise)	L	7.5	±	0.6	7.8	±	1.0	6.8	±	2.2	7.8	±	1.9	0.89	0.12	0.60	0.88
	H	7.3	±	1.6	7.3	±	2.3	7.7	±	1.5	8.1	±	0.5				
Time trial total work (kJ)	L	81.7	±	54.9	124.5	±	73.6	96.1	±	48.2	124.1	±	74.3	0.78	0.07	0.99	0.60
	H	94.5	±	63.8	118.8	±	71.7	88.5	±	83.5	126.5	±	100.0				

(a) MA: mangiferin, Pre-Post: comparison of main effects between 48 h and 15 days, T × t: treatment by time interaction; T × t × d: Treatment × time × dose interaction, L: 50 mg of luteolin and 100 mg mangiferin; H: 100 mg of luteolin and 300 mg mangiferin; MFO: maximal fat oxidation, VO_2_: oxygen uptake, Wmax: power output reached at exhaustion during the incremental exercise, HRmax: maximal heart rate during the incremental exercise, VO_2_max: maximal oxygen uptake, RERmax: respiratory exchange ratio at maximal exercise, V_E_max: pulmonary ventilation at maximal exercise, BFmax: breathing frequency at maximal exercise, P_ET_CO_2_: end-tidal carbon dioxide pressure, P_ET_O_2_: end-tidal oxygen pressure, (n = 12 for all variables). (b) MA: mangiferin, Pre-Post: comparison of main effects between 48 h and 15 days, T × t: treatment by time interaction; T × t × d: Treatment × time × dose interaction, L: 50 mg of luteolin and 100 mg mangiferin; H: 100 mg of luteolin and 300 mg mangiferin; Lact: blood lactate concentration, LT 4 mM: Power output at the Lactate threshold of 4 mM, RPE: rate of perceived exertion, (n = 12 for all variables, except the final time trial n = 11).

**Table 2:** Effects of mangiferin and luteolin botanical extracts on heart rate and pulmonary gas exchange during 15 s all-out sprint performed after ischemia/reperfusion, immediately after the incremental exercise to exhaustion.

		First 15 s Sprint												Second 15 s Sprint															
		Placebo (48 h)			Placebo (15 Days)			MA + Luteolin (48 h)			MA + Luteolin (15 Days)			Placebo (48 h)			Placebo (15 Days)			MA + Luteolin (48 h)			MA + Luteolin (15 Days)			Sprint	Treat	Pre-Post	Sprint × Treat
HR (beats/min)	L	170	±	12	171	±	13	168	±	12	170	±	13	168	±	12	172	±	11	165	±	11	170	±	14	0.36	0.79	0.011	0.74
	H	180	±	15	182	±	10	180	±	14	184	±	13 *	178	±	16	182	±	9	177	±	14	186	±	14 *				
VO_2_ (mL)	L	530	±	121	542	±	101	515	±	105	554	±	127	728	±	160	749	±	98	655	±	76	729	±	94	<0.001	0.010	0.038	0.99
	H	585	±	85	577	±	93	461	±	124	553	±	69	823	±	112	799	±	131	740	±	85	821	±	120				
O_2_ Deficit (mL)	L	164	±	163	219	±	233	130	±	70	195	±	74	28	±	158	51	±	136	38	±	119	41	±	121	<0.001	0.19	0.42	0.94
	H	306	±	87	338	±	106	399	±	175	466	±	58	129	±	142	139	±	90	232	±	249	184	±	53				
V_E_ (L/min)	L	95	±	36	102	±	39	101	±	25	107	±	42	121	±	39	123	±	35	115	±	32	126	±	49	<0.001	0.74	0.025	0.74
	H	119	±	32	122	±	18	104	±	24	119	±	18	138	±	23	150	±	21	134	±	13	155	±	11				
BF (breaths/min)		48	±	13	50	±	14	49	±	8	42	±	9	53	±	13	55	±	13	53	±	11	49	±	11	0.029	0.61	0.52	0.72
		52	±	11	58	±	11	55	±	12	55	±	13	57	±	11	61	±	7 *	59	±	10	62	±	8				
P_ET_CO_2_ (mmHg)	L	29	±	3	30	±	7	28	±	4	29	±	8	31	±	6	24	±	10	30	±	5	28	±	9	0.77	0.91	0.046	0.25
	H	27	±	5	25	±	6	25	±	4	25	±	5	29	±	3	26	±	4 *	27	±	4	27	±	3				
P_ET_O_2_ (mmHg)	L	119	±	4	118	±	7	121	±	4	119	±	8	116	±	6	122	±	8	116	±	5	117	±	10	0.057	0.72	0.101	0.178
	H	121	±	4	122	±	6	124	±	5	123	±	6	118	±	3	120	±	3	120	±	4	120	±	4				

MA: mangiferin, Sprint: differences between sprints, Treat: treatment effect, Pre-Post (time effect): comparison of main effects between 48 h and 15 days, Sprint × treat: Sprint × treatment interaction, L: 50 mg of luteolin and 100 mg mangiferin; H: 100 mg of luteolin and 300 mg mangiferin, VO_2_: oxygen uptake, HR: heart rate, VO_2_: total O_2_ uptake during the sprint, V_E_: pulmonary ventilation, BF: breathing frequency, P_ET_CO_2_: end-tidal carbon dioxide pressure, P_ET_O_2_: end-tidal oxygen pressure, (n = 10 for all variables). Two subjects were eliminated from the statistical analysis due to missing values. * *p* < 0.05 compared with 48 h test in the same condition.

**Table 3:** Effects of mangiferin and luteolin botanical extracts on heart rate and pulmonary gas exchange during the 30-s all-out sprint (Wingate tests) performed after a 30 min recovery period and interspaced by 4 min of unloaded pedaling.

		First 30 s Sprint												Second 30 s Sprint															
		Placebo (48 h)			Placebo (15 Days)			MA + Luteolin (48 h)			MA + Luteolin (15 Days)			Placebo (48 h)			Placebo (15 Days)			MA + Luteolin (48 h)			MA+ Luteolin (15 Days)			Sprint	Treat	Pre-Post	Sprint × Treat
HR (beats/min)	L	164	±	6	164	±	7	161	±	2	162	±	6	165	±	6	165	±	7	164	±	4	166	±	8	0.011	0.92	0.62	0.058
	H	169	±	10	170	±	9	170	±	9	171	±	13	171	±	8	171	±	9	172	±	8	172	±	10				
VO_2_ (mL)	L	1321	±	238	1292	±	239	1182	±	181	1337	±	240	1393	±	196	1392	±	293	1379	±	183	1442	±	203	<0.001	0.59	0.58	0.27
	H	1230	±	246	1287	±	143	1264	±	209	1226	±	181	1435	±	234	1414	±	202	1428	±	257	1377	±	177				
O_2_ Deficit (mL)	L	1566	±	307	1415	±	275	1573	±	358	1421	±	348	1212	±	332	1091	±	269	1141	±	256	1167	±	290	<0.001	0.55	0.58	0.55
	H	1783	±	358	1683	±	276	1794	±	314	1610	±	362	1211	±	295	1291	±	228	1364	±	229	1258	±	277				
V_E_ (L/min)	L	86	±	23	83	±	26	78	±	19	91	±	40	115	±	33	124	±	44	119	±	29	122	±	34	<0.001	0.82	0.49	0.71
	H	98	±	23	102	±	24	101	±	24	99	±	18	134	±	16	135	±	20	137	±	10	136	±	15				
BF (breaths/min)	L	49	±	11	49	±	12	48	±	8	43	±	10	52	±	12	51	±	9	53	±	10	49	±	12	0.001	0.88	0.34	0.108
	H	49	±	10	50	±	11	48	±	15	52	±	16	56	±	11	55	±	9	61	±	11	58	±	10				
P_ET_CO_2_ (mmHg)	L	28	±	2	26	±	4	26	±	6	27	±	5	26	±	4	24	±	6	25	±	5	26	±	5	0.101	0.73	0.71	0.57
	H	23	±	7	23	±	5	24	±	7	24	±	5	23	±	5	23	±	5	22	±	5	23	±	4				
P_ET_O_2_ (mmHg)	L	112	±	4	113	±	5	113	±	9	111	±	8	117	±	5	120	±	6	119	±	5	117	±	6	<0.001	0.626	0.783	0.548
	H	119	±	8	118	±	8	117	±	11	117	±	8	122	±	6	122	±	4	122	±	6	122	±	4				

MA: mangiferin, Sprint: differences between the first and the second sprints, Treat: treatment effect, Pre-Post (time effect): comparison of main effects between 48 h and 15 days, Sprint × treat: Sprint × treatment interaction, L: 50 mg of luteolin and 100 mg mangiferin; H: 100 mg of luteolin and 300 mg mangiferin, HR: heart rate, VO_2_: total O_2_ uptake during the sprint, V_E_: pulmonary ventilation, BF: breathing frequency, P_ET_CO_2_: end-tidal carbon dioxide pressure, P_ET_O_2_: end-tidal oxygen pressure, (n = 10 for all variables). Two subjects were eliminated from the statistical analysis due to occasional missing values. * *p* < 0.05 compared with 48 h test in the same condition.

## Supplementary Materials

The following are available online at http://www.mdpi.com/2072-6643/11/2/344/s1, Table S1: Effects of mangiferin and luteolin botanical extracts on blood biochemistry tests, Table S2: Effects of mangiferin and luteolin botanical extracts on blood hematology tests, Table S3: Effects of mangiferin and luteolin botanical extracts on body mass and cardiorespiratory variables measured at rest.
