# Supplementary material for: Enhancement of Exercise Performance by 48 Hours, and 15-Day Supplementation with Mangiferin and Luteolin in Men
Source: Nutrients. 2019 Feb 6;11(2):344. doi: 10.3390/nu11020344 (PMC6412949; doi:10.3390/nu11020344)
Supplement: Supplementary file 1 [file nutrients-11-00344-s001.zip › Supplementary files/Table S1 FIN (english edits).docx]

| **Table S1**. Effects of mangiferin and luteolin botanical extracts on blood biochemistry tests. | | | | | | | | | | | | | | | | | | | |
| --- | --- | --- | --- | --- | --- | --- | --- | --- | --- | --- | --- | --- | --- | --- | --- | --- | --- | --- | --- |
|  | Control | | | Placebo (48 h) | | | Placebo (15 days) | | | MA + luteolin (48 h) | | | MA + luteolin (15 days) | | | Treatment | Pre-Post | T x t | T x t x d |
| Glucose (mg/dL) | 87.0 | ± | 4.6 | 85.7 | ± | 4.6 | 87.2 | ± | 4.3 | 85.6 | ± | 4.1 | 85.6 | ± | 4.2 | 0.80 | 0.16 | 0.80 | 0.91 |
| Cholesterol (mg/dL) | 152.6 | ± | 22.0 | 144.1 | ± | 19.6 | 142.3 | ± | 22.5 | 146.1 | ± | 23.3 | 144.7 | ± | 22.7 | 0.57 | 0.51 | 0.96 | 0.97 |
| Triglycerides (mg/dL) | 59.8 | ± | 14.2 | 59.8 | ± | 23.1 | 63.1 | ± | 19.9 | 61.0 | ± | 24.0 | 62.8 | ± | 26.7 | 0.93 | 0.71 | 0.91 | 0.31 |
| Uric acid (mg/dL) | 5.1 | ± | 1.0 | 4.6 | ± | 0.8 | 4.5 | ± | 0.7 | 4.8 | ± | 0.9 | 4.6 | ± | 0.7 | 0.27 | 0.45 | 0.55 | 0.16 |
| Creatinine (mg/dL) | 1.0 | ± | 0.1 | 0.9 | ± | 0.1 | 0.9 | ± | 0.1 | 0.9 | ± | 0.1 | 0.9 | ± | 0.1 | 0.51 | 0.24 | 0.57 | 1.0 |
| Hemoglobin (g/dL) | 15.3 | ± | 0.8 | 15.1 | ± | 0.7 | 14.9 | ± | 0.8 | 15.2 | ± | 0.9 | 14.7 | ± | 0.8 | 0.65 | 0.009 | 0.37 | 0.70 |
| Sodium (mEq/L) | 140.9 | ± | 0.6 | 141.6 | ± | 1.5 | 141.2 | ± | 1.2 | 141.1 | ± | 0.7 | 140.7 | ± | 1.2 | 0.2 | 0.27 | 0.83 | 0.55 |
| Potassium (mEq/L) | 4.7 | ± | 0.2 | 4.6 | ± | 0.3 | 4.5 | ± | 0.2 | 4.7 | ± | 0.3 | 4.4 | ± | 0.3 | 0.86 | 0.059 | 0.21 | 0.47 |
| Plasma total Proteins (g/dL) | 7.6 | ± | 0.2 | 7.3 | ± | 0.3 | 6.9 | ± | 0.7 | 7.4 | ± | 0.4 | 7.1 | ± | 0.5 | 0.46 | 0.097 | 0.67 | 0.33 |
| Aspartate transaminase (IU/L) | 26.3 | ± | 6.4 | 25.8 | ± | 10.3 | 25.6 | ± | 13.9 | 24.8 | ± | 8.4 | 24.6 | ± | 7.3 | 0.71 | 0.92 | 0.98 | 0.59 |
| Alanine transaminase (IU/L) | 34.3 | ± | 12.9 | 35.4 | ± | 23.8 | 35.3 | ± | 30.8 | 35.3 | ± | 26.7 | 35.3 | ± | 21.8 | 0.99 | 0.99 | 0.99 | 0.26 |
| Gamma G.T. (IUL/L) | 21.9 | ± | 11.5 | 19.8 | ± | 10.6 | 19.3 | ± | 8.8 | 21.3 | ± | 10.8 | 20.5 | ± | 10.2 | 0.15 | 0.34 | 0.85 | 0.51 |
| Alkaline phosphatase (IU/L) | 66.9 | ± | 17.3 | 64.0 | ± | 16.8 | 64.9 | ± | 19.0 | 66.8 | ± | 20.0 | 65.8 | ± | 15.4 | 0.28 | 1.0 | 0.66 | 0.20 |
| Bilirubin total (mg/dL) | 1.1 | ± | 0.9 | 1.1 | ± | 1.0 | 1.0 | ± | 0.8 | 1.1 | ± | 0.8 | 1.1 | ± | 0.9 | 0.89 | 0.34 | 0.40 | 0.58 |
| Bilirubin direct (mg/dL) | 0.1 | ± | 0.0 | 0.1 | ± | 0.0 | 0.1 | ± | 0.1 | 0.2 | ± | 0.2 | 0.2 | ± | 0.2 | 0.15 | 0.43 | 0.45 | 0.28 |
| Bilirubin indirect (mg/dL) | 0.9 | ± | 0.9 | 1.0 | ± | 1.0 | 0.9 | ± | 0.7 | 0.9 | ± | 0.6 | 0.9 | ± | 0.8 | 0.47 | 0.15 | 0.59 | 0.39 |
| CPK (IU/L) | 217.1 | ± | 209.1 | 189.3 | ± | 207.6 | 145.7 | ± | 109.1 | 136.2 | ± | 83.3 | 134.3 | ± | 82.5 | 0.39 | 0.42 | 0.53 | 0.86 |
| MA: mangiferin; Pre-Post: comparison of main effects between 48 h and 15 days, T x t: treatment by time interaction; T x t x d: Treatment x time x dose interaction; Gamma G.T.: gamma-glutamyl transpeptidase. | | | | | | | | | | | | | | | | | | | |

| **Table S2**. Effects of *Mangifera Indica* L. leaf extract in combination with luteolin in blood hematology tests. | | | | | | | | | | | | | | | | | | | |
| --- | --- | --- | --- | --- | --- | --- | --- | --- | --- | --- | --- | --- | --- | --- | --- | --- | --- | --- | --- |
| Hemogram | Control | | | Placebo (48 h) | | | Placebo (15 days) | | | MLE + Luteolin (48 h) | | | MLE + Luteolin (15 days) | | | Treatment | Pre-Post | T x t | T x t x d |
| Erythrocytes (10^6^/μL) | 5.3 | ± | 0.4 | 5.2 | ± | 0.3 | 5.1 | ± | 0.3 | 5.2 | ± | 0.3 | 5.0 | ± | 0.3 | 0.575 | 0.008 | 0.583 | 0.596 |
| Hematocrit (%) | 46.2 | ± | 2.2 | 45.3 | ± | 1.9 | 44.5 | ± | 2.1 | 45.5 | ± | 2.5 | 44.1 | ± | 2.0 | 0.643 | 0.002 | 0.354 | 0.495 |
| MCV (fL) | 88.0 | ± | 3.3 | 87.8 | ± | 3.4 | 87.8 | ± | 3.0 | 87.9 | ± | 3.2 | 87.8 | ± | 3.2 | 0.749 | 0.585 | 0.864 | 0.957 |
| MCH (pg) | 29.2 | ± | 1.0 | 29.3 | ± | 0.9 | 29.4 | ± | 1.0 | 29.3 | ± | 0.9 | 29.3 | ± | 0.9 | 0.804 | 0.723 | 0.701 | 0.384 |
| MCHC (g/dL) | 33.2 | ± | 0.6 | 33.4 | ± | 0.6 | 33.5 | ± | 0.7 | 33.3 | ± | 0.7 | 33.4 | ± | 0.7 | 0.633 | 0.332 | 0.993 | 0.346 |
| Platelets (10^3^/μL) | 244.3 | ± | 32.8 | 240.0 | ± | 37.2 | 244.8 | ± | 39.5 | 244.3 | ± | 51.8 | 252.3 | ± | 46.2 | 0.658 | 0.511 | 0.853 | 0.137 |
| Leukocytes (10^3^/μL) | 6.5 | ± | 1.1 | 6.3 | ± | 1.1 | 7.0 | ± | 1.3 | 6.6 | ± | 1.4 | 6.8 | ± | 1.0 | 0.075 | 0.121 | 0.401 | 0.323 |
| Neutrophils (10^3^/μL) | 3.3 | ± | 0.8 | 3.3 | ± | 0.8 | 3.7 | ± | 1.2 | 3.5 | ± | 1.2 | 3.5 | ± | 0.8 | 0.941 | 0.294 | 0.451 | 0.190 |
| Eosinophils (10^3^/μL) | 0.2 | ± | 0.1 | 0.3 | ± | 0.1 | 0.3 | ± | 0.2 | 0.3 | ± | 0.2 | 0.3 | ± | 0.2 | 0.966 | 0.916 | 0.538 | 0.898 |
| Lymphocytes (10^3^/μL) | 2.2 | ± | 0.5 | 2.2 | ± | 0.5 | 2.4 | ± | 0.5 | 2.2 | ± | 0.4 | 2.4 | ± | 0.6 | 0.886 | 0.032 | 0.832 | 0.437 |
| Monocytes (10^3^/μL) | 0.6 | ± | 0.1 | 0.5 | ± | 0.1 | 0.6 | ± | 0.2 | 0.6 | ± | 0.1 | 0.6 | ± | 0.1 | 0.942 | 0.059 | 0.437 | 0.209 |
| Basophils (10^3^/μL) | 0.0 | ± | 0.0 | 0.0 | ± | 0.0 | 0.0 | ± | 0.0 | 0.0 | ± | 0.0 | 0.0 | ± | 0.0 | 0.774 | 0.239 | 0.239 | 0.868 |
| Pre-Post: comparison of main effects between 48h and 15 days, Tx t: treatment by time interaction; T x t x d: Treatment x time x dose interaction | | | | | | | | | | | | | | | | | | | |
