# Supplementary material for: Enhancement of Exercise Performance by 48 Hours, and 15-Day Supplementation with Mangiferin and Luteolin in Men
Source: Nutrients. 2019 Feb 6;11(2):344. doi: 10.3390/nu11020344 (PMC6412949; doi:10.3390/nu11020344)
Supplement: Supplementary file 1 [file nutrients-11-00344-s001.zip › Supplementary files/Table S2 FIN (english edits).docx]

| **Table S2**. Effects of mangiferin and luteolin botanical extracts on blood hematology tests. | | | | | | | | | | | | | | | | | | | |
| --- | --- | --- | --- | --- | --- | --- | --- | --- | --- | --- | --- | --- | --- | --- | --- | --- | --- | --- | --- |
|  | Control | | | Placebo (48 h) | | | Placebo (15 days) | | | MA + luteolin (48 h) | | | MA + luteolin (15 days) | | | Treatment | Pre-Post | T x t | T x t x d |
| Erythrocytes (10^6^/μL) | 5.3 | ± | 0.4 | 5.2 | ± | 0.3 | 5.1 | ± | 0.3 | 5.2 | ± | 0.3 | 5.0 | ± | 0.3 | 0.58 | 0.008 | 0.58 | 0.6 |
| Hematocrit (%) | 46.2 | ± | 2.2 | 45.3 | ± | 1.9 | 44.5 | ± | 2.1 | 45.5 | ± | 2.5 | 44.1 | ± | 2.0 | 0.64 | 0.01 | 0.60 | 0.5 |
| MCV (fL/cell) | 88.0 | ± | 3.3 | 87.8 | ± | 3.4 | 87.8 | ± | 3.0 | 87.9 | ± | 3.2 | 87.8 | ± | 3.2 | 0.75 | 0.59 | 0.86 | 0.96 |
| MCH (pg/cell) | 29.2 | ± | 1.0 | 29.3 | ± | 0.9 | 29.4 | ± | 1.0 | 29.3 | ± | 0.9 | 29.3 | ± | 0.9 | 0.80 | 0.72 | 0.70 | 0.38 |
| MCHC (g/dL) | 33.2 | ± | 0.6 | 33.4 | ± | 0.6 | 33.5 | ± | 0.7 | 33.3 | ± | 0.7 | 33.4 | ± | 0.7 | 0.63 | 0.33 | 0.99 | 0.35 |
| Platelets (10^3^/μL) | 244.3 | ± | 32.8 | 240.0 | ± | 37.2 | 244.8 | ± | 39.5 | 244.3 | ± | 51.8 | 252.3 | ± | 46.2 | 0.66 | 0.51 | 0.85 | 0.14 |
| Leukocytes (10^3^/μL) | 6.5 | ± | 1.1 | 6.3 | ± | 1.1 | 7.0 | ± | 1.3 | 6.6 | ± | 1.4 | 6.8 | ± | 1.0 | 0.94 | 0.14 | 0.401 | 0.32 |
| Neutrophils (10^3^/μL) | 3.3 | ± | 0.8 | 3.3 | ± | 0.8 | 3.7 | ± | 1.2 | 3.5 | ± | 1.2 | 3.5 | ± | 0.8 | 0.94 | 0.29 | 0.45 | 0.19 |
| Eosinophils (10^3^/μL) | 0.2 | ± | 0.1 | 0.3 | ± | 0.1 | 0.3 | ± | 0.2 | 0.3 | ± | 0.2 | 0.3 | ± | 0.2 | 0.97 | 0.92 | 0.54 | 0.9 |
| Lymphocytes (10^3^/μL) | 2.2 | ± | 0.5 | 2.2 | ± | 0.5 | 2.4 | ± | 0.5 | 2.2 | ± | 0.4 | 2.4 | ± | 0.6 | 0.97 | 0.035 | 0.70 | 0.43 |
| Monocytes (10^3^/μL) | 0.6 | ± | 0.1 | 0.5 | ± | 0.1 | 0.6 | ± | 0.2 | 0.6 | ± | 0.1 | 0.6 | ± | 0.1 | 1.0 | 0.075 | 0.3 | 0.3 |
| Basophils (10^3^/μL) | 0.0 | ± | 0.0 | 0.0 | ± | 0.0 | 0.0 | ± | 0.0 | 0.0 | ± | 0.0 | 0.0 | ± | 0.0 | 0.77 | 0.24 | 0.24 | 0.87 |
| MA: mangiferin, Pre-Post: comparison of main effects between 48 h and 15 days, T x t: treatment by time interaction, T x t x d: Treatment x time x dose interaction. | | | | | | | | | | | | | | | | | | | |
