# Supplementary material for: Enhancement of Exercise Performance by 48 Hours, and 15-Day Supplementation with Mangiferin and Luteolin in Men
Source: Nutrients. 2019 Feb 6;11(2):344. doi: 10.3390/nu11020344 (PMC6412949; doi:10.3390/nu11020344)
Supplement: Supplementary file 1 [file nutrients-11-00344-s001.zip › Supplementary files/Table S3 FIN (english edits).docx]

| **Table S3**. Effects of mangiferin and luteolin botanical extracts on body mass and cardiorespiratory variables measured at rest. | | | | | | | | | | | | | | | | | | |
| --- | --- | --- | --- | --- | --- | --- | --- | --- | --- | --- | --- | --- | --- | --- | --- | --- | --- | --- |
|  |  | Placebo (48 h) | | | Placebo (15 days) | | | MA + Luteolin (48 h) | | | MA + Luteolin (15 days) | | | Treatment | Pre-Post | T x t | T x t x d |  |
| Body weight (kg) | L | 73.0 | ± | 9.2 | 73.2 | ± | 9.0 | 73.7 | ± | 10.3 | 73.8 | ± | 10.4 | 0.26 | 0.42 | 0.26 | 0.43 |  |
|  | H | 78.5 | ± | 12.0 | 78.9 | ± | 12.1 | 79.2 | ± | 12.3 | 79.0 | ± | 12.0 |  |  |  |  |  |
| Whole-body lean mass (kg) | L | 56.080 | ± | 4.645 | 56.214 | ± | 5.101 | 56.188 | ± | 5.336 | 55.978 | ± | 5.258 | 0.91 | 0.70 | 0.068 | 0.30 |  |
|  | H | 57.336 | ± | 8.207 | 57.845 | ± | 8.325 | 58.040 | ± | 8.457 | 57.426 | ± | 8.226 |  |  |  |  |  |
| Resting HR (beats/min) | L | 60 | ± | 5 | 62 | ± | 8 | 59 | ± | 6 | 58 | ± | 7 | 0.26 | 0.58 | 0.77 | 0.74 |  |
|  | H | 68 | ± | 8 | 69 | ± | 7 | 66 | ± | 11 | 68 | ± | 6 |  |  |  |  |  |
| Systolic BP (mmHg) | L | 124.1 | ± | 4.9 | 121.0 | ± | 3.3 | 119.3 | ± | 5.9 | 124.2 | ± | 5.5 | 0.7 | 0.87 | 0.23 | 0.34 |  |
|  | H | 120.7 | ± | 12.8 | 119.9 | ± | 8.3 | 119.3 | ± | 10.4 | 119.6 | ± | 5.3 |  |  |  |  |  |
| Diastolic BP (mmHg) | L | 61.7 | ± | 3.2 | 61.5 | ± | 5.5 | 61.8 | ± | 7.5 | 58.7 | ± | 6.6 | 0.40 | 0.41 | 0.9 | 0.23 |  |
|  | H | 64.1 | ± | 8.6 | 62.2 | ± | 6.9 | 61.1 | ± | 7.3 | 62.7 | ± | 6.3 |  |  |  |  |  |
| Resting VO_2_ (mL/min) | L | 307 | ± | 46 | 296 | ± | 53 | 300 | ± | 45 | 308 | ± | 48 | 0.60 | 0.19 | 0.18 | 0.90 |  |
|  | H | 325 | ± | 35 | 301 | ± | 49 | 316 | ± | 42 | 316 | ± | 37 |  |  |  |  |  |
| RER | L | 0.82 | ± | 0.04 | 0.85 | ± | 0.05 | 0.83 | ± | 0.06 | 0.87 | ± | 0.08 | 0.71 | 0.025 | 0.58 | 0.82 |  |
|  | H | 0.82 | ± | 0.05 | 0.84 | ± | 0.06 | 0.81 | ± | 0.04 | 0.84 | ± | 0.06 |  |  |  |  |  |
| Resting energy expenditure (Kcal/d) | L | 2207 | ± | 323 | 2141 | ± | 366 | 2157 | ± | 316 | 2238 | ± | 331 | 0.57 | 0.34 | 0.17 | 0.92 |  |
|  | H | 2332 | ± | 247 | 2170 | ± | 340 | 2268 | ± | 284 | 2275 | ± | 240 |  |  |  |  |  |
| V_E_ (L/min) | L | 7.8 | ± | 1.0 | 7.9 | ± | 1.1 | 7.8 | ± | 1.3 | 8.3 | ± | 1.0 | 0.39 | 0.20 | 0.28 | 0.82 |  |
|  | H | 8.8 | ± | 1.4 | 8.8 | ± | 1.0 | 8.7 | ± | 1.2 | 9.2 | ± | 1.3 |  |  |  |  |  |
| P_ET_CO_2_ (mmHg) | L | 39.4 | ± | 0.6 | 39.3 | ± | 1.4 | 38.6 | ± | 1.5 | 38.7 | ± | 0.9 | 0.14 | 0.75 | 0.34 | 0.60 |  |
|  | H | 37.9 | ± | 1.3 | 37.7 | ± | 1.5 | 37.0 | ± | 1.2 | 37.6 | ± | 1.1 |  |  |  |  |  |
| BF (breaths/min) | L | 12.1 | ± | 3.6 | 13.1 | ± | 4.3 | 12.9 | ± | 3.7 | 13.0 | ± | 3.8 | 0.41 | 0.03 | 0.98 | 0.38 |  |
|  | H | 13.3 | ± | 2.7 | 14.0 | ± | 1.7 | 13.2 | ± | 1.8 | 14.7 | ± | 3.3 |  |  |  |  |  |
| MA: mangiferin, Pre-Post: comparison of main effects between 48 h and 15 days, T x t: treatment by time interaction, T x t x d: Treatment x time x dose interaction, L: 50 mg of luteolin and 100 mg Mangiferin; H: 100 mg of luteolin and 300 mg Mangiferin; RER: respiratory exchange ratio, V_E_: pulmonary ventilation, P_ET_CO_2_: end-tidal carbon dioxide pressure, BF: breathing frequency. N=12 for all variables. | | | | | | | | | | | | | | | | | |  |
